# *Chlamydia abortus* Pmp18.1 Induces IL-1β Secretion by TLR4 Activation through the MyD88, NF-κB, and Caspase-1 Signaling Pathways

**DOI:** 10.3389/fcimb.2017.00514

**Published:** 2017-12-18

**Authors:** Qing Pan, Qiang Zhang, Jun Chu, Roshan Pais, Shanshan Liu, Cheng He, Francis O. Eko

**Affiliations:** ^1^Department of Microbiology, Biochemistry and Immunology, Morehouse School of Medicine, Atlanta, GA, United States; ^2^Key Lab of Animal Epidemiology and Zoonosis of Ministry of Agriculture, College of Veterinary Medicine, China Agricultural University, Beijing, China

**Keywords:** *Chlamydia abortus*, dendritic cells, Pmp18D, TLR4, NF-κB signaling, IL-1β

## Abstract

The polymorphic membrane protein D (Pmp18D) is a 160-kDa outer membrane protein that is conserved and plays an important role in *Chlamydia abortus* pathogenesis. We have identified an N-terminal fragment of Pmp18D (designated Pmp18.1) as a possible subunit vaccine antigen. In this study, we evaluated the vaccine potential of Pmp18.1 by investigating its ability to induce innate immune responses in dendritic cells and the signaling pathway(s) involved in rPmp18.1-induced IL-1β secretion. We next investigated the immunomodulatory impact of VCG, in comparison with the more established Th1-promoting adjuvants, CpG and FL, on rPmp18.1-mediated innate immune activation. Finally, the effect of siRNA targeting TLR4, MyD88, NF-κB p50, and Caspase-1 mRNA in DCs on IL-1β cytokine secretion was also investigated. Bone marrow-derived dendritic cells (BMDCs) were stimulated with rPmp18.1 in the presence or absence of VCG or CpG or FL and the magnitude of cytokines produced was assessed using a multiplex cytokine ELISA assay. Expression of costimulatory molecules and Toll-like receptors (TLRs) was analyzed by flow cytometry. Quantitation of intracellular levels of myeloid differentiation factor 88 (MyD88), nuclear factor kappa beta (NF-κB p50/p65), and Caspase-1 was evaluated by Western immunoblotting analysis while NF-κB p65 nuclear translocation was assessed by confocal microscopy. The results showed DC stimulation with rPmp18.1 provoked the secretion of proinflammatory cytokines and upregulated expression of TLRs and co-stimulatory molecules associated with DC maturation. These responses were significantly (*p* ≤ 0.001) enhanced by VCG but not CpG or FL. In addition, rPmp18.1 activated the expression of MyD88, NF-κB p50, and Caspase-1 as well as the nuclear expression of NF-κB p65 in treated DCs. Furthermore, targeting TLR4, MyD88, NF-κB p50, and Caspase-1 mRNA in BMDCs with siRNA significantly reduced their expression levels, resulting in decreased IL-1β cytokine secretion, strongly suggesting their involvement in the rPmp18.1-induced IL-1β cytokine secretion. Taken together, these results indicate that *C. abortus* Pmp18.1 induces IL-1β secretion by TLR4 activation through the MyD88, NF-κB as well as the Caspase-1 signaling pathways and may be a potential *C. abortus* vaccine candidate. The vaccine potential of Pmp18.1 will subsequently be evaluated in an appropriate animal model, using VCG as an immunomodulator, following immunization and challenge.

## Introduction

Infection with *Chlamydia abortus* (*C. abortus*), the causative agent of ovine enzootic abortion (OEA) in sheep, pigs, and goats, leads to considerable economic losses worldwide (Reddy et al., 1997; Longbottom et al., 2002). In addition, it poses a zoonotic risk to pregnant women (Longbottom and Coulter, 2003). The infection, which is acquired through ingestion or inhalation of *C. abortus*-infected material (Wilsmore et al., 1984; Dawson et al., 1986), is characterized by acute placentitis and abortion usually occurs before the end of pregnancy. Natural infection is often asymptomatic until pregnancy occurs when the organism invades the placenta, ultimately causing abortion (Gutierrez et al., 2011; Longbottom et al., 2013). Many zoonotic infections are also asymptomatic and lead to the development of complications, such as septicemia, extemporaneous fetal abortion, and stillbirth (Walder et al., 2005; Baud et al., 2008). Thus, development of a safe vaccine that can protect against infection would be the best approach to control infections and the associated complications.

Current live attenuated vaccines, while effective against infection, are unsafe as indicated by their implication in cases of abortion (Wheelhouse et al., 2010). The vaccines are expensive and challenging to manufacture in large quantities, as production is hazardous and labor-intensive. Besides, it is difficult to monitor vaccination practices due to challenges in serologically distinguishing infected from vaccinated animals (Gerber et al., 2007). Inactivated vaccines are only marginally protective (Longbottom and Livingstone, 2006), highlighting the need to search for alternative vaccine development strategies. The superior safety profiles of subunit vaccines have made them more attractive than traditional vaccines, which are based on live attenuated or whole inactivated pathogens.

Since vaccines based on subunit antigens are generally poorly immunogenic, they usually require co-administration with adjuvants for induction of optimal protective immunity (Moyle and Toth, 2013; Savelkoul et al., 2015). In this respect, a number of adjuvants have previously been tested with different chlamydial subunit antigens (reviewed in Yu et al., 2016), including monophosphoryl lipid A (MPLA), ISCOM (AbISCO-100), CpG-ODN1826, and Montanide ISA720 (Yu et al., 2010, 2012; Tifrea et al., 2013). Also, the CpG motif, which is the agonist of Toll-like receptor (TLR) 9, is a well-known Th1-stimulating adjuvant (Cheng et al., 2009) and the Fms-like tyrosine kinase 3 Ligand (Flt3L; FL), a molecule that increases the number of immune cells is a proven dendritic cell (DC)-targeting adjuvant (Fukuyama et al., 2011). A combination of CpG and FL used as a mucosal adjuvant was shown to enhance the immune responses of co-delivered antigens (Fukuyama et al., 2011; Asanuma et al., 2012). In addition, the *Vibrio cholerae* ghost (VCG) platform has been demonstrated to be an effective immunomodulator for both subunit and inactivated chlamydial antigens (Eko et al., 2014). VCG are empty bacterial cell envelopes derived from *V. cholerae* cells by protein E-mediated lysis. VCG are devoid of cytoplasmic contents but possess the functional and antigenic determinants of the envelope complex with their living counterparts (Eko et al., 2003).

Dendritic cells (DCs) are a group of professional APCs, which initiate and control antigen-specific immune responses when stimulated with pathogen associated molecular patterns (PAMPs) (Banchereau and Steinman, 1998). Immature DCs (iDCs) are characterized by their high endocytic ability and low membrane expression of MHC II molecules. Pathogen-associated molecules, such as Toll-like receptor (TLR) agonists, activate iDCs to undergo phenotypic changes that lead to the acquisition of a “mature” phenotype (Steinman et al., 2000; Young et al., 2007). When activated, DCs migrate from peripheral tissues to draining lymph nodes where they express cell surface and secreted molecules associated with immune regulation (Banchereau and Steinman, 1998). Unlike iDCs, mature DCs are characterized by low endocytosis, high migration into lymphoid tissue, expression of high levels of MHC-I/II, co-stimulatory molecules, and high secretion of cytokines (Villadangos and Schnorrer, 2007). TLRs/MyD88/IRAK/TRAF/NF-κB signaling pathway is involved in innate and adaptive immunity (Cook et al., 2004; Ohnishi et al., 2009). Nucleotide-binding and oligomerization domain (NOD)-like receptors (NLRs), which are highly conserved cytosolic pattern recognition receptors, together with toll-like receptors, play a critical role in induction of innate immune responses and inflammation (Corridoni et al., 2014). Some NLRs, such as NOD1 and NOD2, drive the activation of mitogen-activated protein kinase and the transcription factor, nuclear factor kappa B (NF-κB) while others induce caspase-1 activation through the assembly of inflammasomes, resulting in pro-inflammatory cytokine secretion and consequent inflammatory responses (Corridoni et al., 2014).

The polymorphic membrane proteins (Pmps) consisting of 18 pmp genes belong to a family of proteins, which resemble the type V or autotransporter secretion system (Kiselev et al., 2009; Tan et al., 2009). Among these, the Pmp18D is a 160 kDa highly conserved and immunogenic outer membrane protein that plays an important role in *C. abortus* pathogenesis. We have identified an N-terminal fragment of Pmp18D (designated Pmp18.1) as a possible subunit vaccine antigen. In this study, we investigated the ability of Pmp18.1 with or without VCG to induce innate immune responses in dendritic cells and the signaling pathway(s) involved in rPmp18.1-induced IL-1β secretion. We showed that rPmp18.1 induced innate immune responses in DCs that were significantly enhanced by VCG. In addition, rPmp18.1 activated the expression of MyD88, NF-κB p50, and Caspase-1, and the nuclear expression of NF-κB p65 in treated DCs. Furthermore, inhibition of these molecules by siRNA targeting significantly reduced their expression levels, resulting in decreased IL-1β cytokine secretion. These results strongly suggest the involvement of MyD88, NF-κB, and Caspase-1 in the rPmp18.1-induced IL-1β cytokine secretion. Taken together, these data indicate that *C. abortus* Pmp18.1 induces IL-1β secretion by TLR4 activation through the MyD88, NF-κB, and Caspase-1 signaling pathways and is a potential *C. abortus* vaccine candidate. The vaccine efficacy of Pmp18.1 will subsequently be evaluated in an animal model following immunization and challenge.

## Materials and methods

### Ethics statement

This study followed the recommendations stipulated in the Guide for the Care and Use of Laboratory Animals of the National Institutes of Health. The Institutional Animal Care and Use Committee (IACUC) of Morehouse School of Medicine (MSM) approved the study protocol (Protocol Number: 16-15). Female C57BL/6J mice (6 to 7-week-old, stock number 000664) obtained from The Jackson Laboratory (Bar Harbor, ME) were used in this study. Mice were allowed to acclimatize for 10 days in the MSM animal facility prior to experimentation.

### *Chlamydia abortus*, antigens, and reagents

*C. abortus* strains CP16 (Chinese Institute of Veterinary Drug Control, Beijing, China) and B577 (ATCC^®^ VR-656™ (obtained from Dr. Bernhard Kaltenboeck, Auburn University, Alabama) were propagated and purified in Buffalo Green Monkey Kidney (BGMK) cells (ATCC cat# PTA-4594) as previously described (Li et al., 2005). Organisms were purified as elementary bodies (EBs) and stored in aliquots at −80°C until use. *C. abortus* antigen was prepared by UV-inactivation of EBs for 3 h followed by sonication using a Sonic Dismembrator Model 505 (Thermo Fisher Scientific, Rockford, IL). VCG were generated by genetic inactivation following protein E-mediated lysis as described previously (Eko et al., 2000). VCG preparations were stored at room temperature until use. Purified mouse Fms-like tyrosine kinase 3 (Flt3) ligand (FL) was obtained from R&D Systems (Minneapolis, MN), CpG 1826 ODN was obtained from InvivoGen (San Diego, CA) and lipopolysaccharide (LPS) was purchased from Sigma (St. Louis, MO).

### Purification of recombinant Pmp18.1 (rPmp18.1)

An N-terminal fragment of the Pmp18D gene (1,317 bp) amplified from the genomic DNA of *C. abortus* strain P16 (GenBank: FJ755794.1) was cloned into vector pET-32a by means of restriction sites incorporated into the primer sets. The generated plasmid, designated pET-18.1 was transformed into *E. coli* BL21 (DE3) (Catalog number: C600003) obtained from Invitrogen (California, USA). Purification of rPmp18.1 was by the Ni-NTA Purification System (Invitrogen, California, USA) according to the manufacturer's instructions. Detoxi-Gel™ Endotoxin Removing Gel (Thermo Fisher Scientific, Rockford, IL) was used to remove contaminating endotoxin. The concentration of remaining endotoxin was < 0.05 EU/mg protein as determined by the Pierce LAL Chromogenic Endotoxin Quantitation Kit (Thermo Fisher Scientific, Rockford, IL). Protein concentration was quantified by the Pierce™ BCA Protein Assay Kit (Thermo Scientific, Illinois), adjusted to 500 μg/ml and stored at −80°C. Immunoblotting analysis of protein expression was performed as described previously (Eko et al., 2011a) using purified rabbit anti-Pmp18D polyclonal antibody.

### Dendritic cell isolation and culture

Immature BMDCs were generated from the bone marrow of 6 week-old C57BL/6 mice by a standard methods (Inaba et al., 2001). Briefly, the marrow cells were cultured in Iscove's Modified Dulbecco's Medium (IMDM) containing the following supplements; 10% FCS, 4 mM L-glutamine, 10 U/ml penicillin, 100 μg/ml streptomycin, 0.5 mM 2-ME 10 ng/ml GM-CSF (PeproTech Inc., NJ), and 10 ng/ml IL-4 (PeproTech Inc., NJ) at 37°C and 5% CO_2_. On day 3, fresh IL-4 and GM-CSF was added to the culture. After 7 days, the loosely adherent and non-adherent cells were harvested, washed, and resuspended in supplemented IMDM.

### Fluorescent antibody staining and flow cytometry

BMDCs (5 × 10^5^ cells per well) were incubated with Pmp18.1 (10 μg/ml) in a 24-well plate with or without VCG (100 μg), CpG (10 μg), or Flt3L (FL; 150 ng) in supplemented IMDM for 24 h at 37°C in 5% CO_2_. LPS (200 ng/ml) was included as a positive control while cultures incubated with culture medium alone were used as negative controls. Antigen-pulsed DC cultures were incubated with phycoerythrin (PE) or Fluorescein isothiocyanate (FITC) conjugated monoclonal antibodies against DC surface markers (CD14, CD40, CD80, CD86, and 1Ab), Toll-like receptors (TLRs) (TLR2, TLR4, TLR5), Nod-like receptor pyrin domain containing 3 (NLRP3) and CCR7 or isotype-matched controls (Pharmingen, San Diego, CA) in FACS buffer (2% FBS; Thermo Fisher Scientific, Rockford, IL) in PBS for 30 min on ice. To reduce non-specific Fc receptor mediated binding, Mouse BD Fc Block (purified rat anti-mouse CD16/CD32; Clone 2.4G2) was added to the cells prior to staining. After washing in FACS buffer, cells were fixed with 2% paraformaldehyde (Thermo Fisher Scientific, Rockford, IL), washed again and analyzed by flow cytometry on a BD Accuri C6 Flow Cytometer in combination with the BD Accuri C6 Software (BD Biosciences, San Jose, CA). Gating was performed on CD11c+ cells.

### Multiplex array for quantitation of cytokines secreted by antigen-pulsed DCs

Twenty four-hour culture supernatants of BMDCs pulsed with Pmp18.1 with or without VCG or CpG or FL were collected and used for simultaneous measurements of 13 mouse cytokines, (13-Plex Group I customized (catalog number L60000F7MP; IL-17A and GM-CSF were replaced with IL-4 and Eotaxin) using the Bio-Plex Pro™ mouse cytokine bead ELISA assay and the Bio-Plex Manager software (Bio-Rad, Hercules, CA) essentially according to the manufacturer's instructions. The mean and *SD* of five replicate cultures were calculated and cytokines were expressed in pg/ml as means and standard deviations. The experiment was repeated twice.

### Evaluation of naive T cell proliferation

We used the DC-naïve T cell proliferation assay to evaluate the ability of Pmp18.1 to elicit helper CD4+ T cell proliferation. Splenocytes obtained from spleens of naïve mice using the gentleMACS Dissociator (Miltenyi Biotech, Auburn, CA) were resuspended in PBS containing BSA and EDTA. Purification of CD4+ T cells was achieved by positive selection using the MidiMACS system and labeled CD4 (L3T4) mouse microbeads (Miltenyi Biotech, Auburn, CA). Purity of the CD4+ T cells was analyzed by flow cytometry and shown to be at least >95%. Purified naïve CD4^+^ T cells (2 × 10^5^ cells/well) were cultured in 96-well plates (Corning Glass Work, Acton, MA) with BMDCs (2 × 10^5^ cells/well) and either Pmp18.1 (10 μg/ml) or Pmp18.1 plus VCG (100 μg) in 200 μl of C-RPMI medium supplemented with GM-CSF at 37°C in 5% CO_2_. Control cultures containing DCs and naïve T cells without antigen served as internal control. After 7 days of culture, T cell proliferation was assessed essentially as described previously (Eko et al., 2011a) using the 5-Bromo-2′-deoxy-uridine (BrdU) cell proliferation assay (Roche Molecular Biochemicals, Indianapolis, IN). Absorbance values were read at 450 nm and the ratio between stimulated and non-stimulated cells (stimulation index, SI) was then calculated.

### Preparation of whole-cell, cytoplasmic, and nuclear extracts

BMDCs were incubated with Pmp18.1 (10 μg/ml) for 1, 2, or 24 h. Preparation of whole cell lysates was essentially as previously described (Chou et al., 2013). Cells were lysed in ice cold RIPA Buffer composed of 250 mM Tris-HCl, 750 mM NaCl, 5% NP-40, 2.5% Sodium deoxycholate and 0.5% SDS at pH 7.4 (Boston BioProducts Inc., Ashland, MA) by sonication using a Sonic Dismembrator Model 505 (Thermo Fisher Scientific, Rockford, IL). Soluble extracts were obtained by centrifuging the supernatants at 15,000 g for 10 min. The cytosolic and nuclear extracts were prepared by lysing Pmp18.1-pulsed DCs with ice cold Buffer A (containing 20 mM HEPES, pH 7.0, 10 mM KCl, 2 mM MgCl_2_, 0.5% NP-40, 1 mM NaF, 1 mM Na_3_VO_4_, 1 mM PMSF, 1 μg/ml aprotinin), homogenized in a PowerGen 500 homogenizer (Thermo Fisher Scientific, Rockford, IL) and then centrifuged at 1,500 g for 10 min. The supernatant constituted the cytosolic fraction. The pellet (nuclear fraction) was washed with cold PBS, lysed in NETN buffer (20 mM Tris at pH 8.0, 150 mM NaCl, 1 mM EDTA, 0.5% NP-40, 1 mM NaF, 1 mM Na_3_VO_4_, 1 mM PMSF, 1 μg/ml aprotinin) (Bethyl Laboratories, Inc. (Montgomery, TX) by sonication and the supernatant containing the nuclear fraction was obtained by centrifugation at 12,000 g for 20 min. The protein concentration was determined using the Pierce BCA Assay Kit (Thermo Fisher Scientific, Rockford, IL).

### Expression of MyD88, NFκB, and caspase-1 by western immunoblotting analysis

About 40 μg/well of each protein extract was separated on a 10% SDS-PAGE and transferred to a 0.45 μm nitrocellulose membrane (Bio-Rad, Hercules, CA). Membranes were blocked with 5% non-fat dry milk solution (catalog number 9999) (Cell Signaling Technology, Danvers, MA) at room temperature for 1 h and reacted with the corresponding protein antibodies at 4°C overnight: myeloid differentiation primary response gene 88 (MyD88) (HFL-296, polyclonal; catalog number sc-11356), nuclear factor kappa B (NF-κB) p50 (H-119, polyclonal; catalog number sc-7178), NFκB p65 (F-6, monoclonal; catalog number sc-8008), Caspase-1 (14F468, monoclonal; catalog number sc-56036), GAPDH (FL-335, polyclonal; catalog number sc-25778) (Santa Cruz, Dallas, TX). After washing five times with PBST, the membrane was incubated with HRP-conjugated anti-mouse or anti-rabbit secondary antibody (Southern Biotech, Birmingham, AL) at room temperature for 1 h. Proteins were detected by addition of the Pierce™ ECL Western Blotting Substrate and visualized using the ImageQuant LAS-4000 (Thermo Fisher Scientific, Rockford, IL). The protein levels, normalized to glutaraldehyde phosphate dehydrogenase (GAPDH), were quantified by Image J software (National Institutes of Health, Bethesda, MD).

### Confocal microscopy

DCs were seeded in an eight-well microtiter plate, cultured with or without Pmp18.1 for 24 h, harvested by digestion with accutase detachment solution (Thermo Fisher Scientific, Rockford, IL) and centrifuged at 1,500 g for 10 min. After washing thrice with FACS Buffer, cells were fixed and permeabilized using BD Cytofix/Cytoperm™ Plus Fixation/Permeabilization Kit with BD GolgiStop™ according to the manufacture's protocol. The permeabilized cells were stained for 30 min on ice with mouse anti-NF-κB p65 monoclonal antibody (Santa Cruz, Dallas, Texas) washed with FACS Buffer, incubated with FITC-conjugated (green) goat anti-mouse IgG secondary antibody (BD Pharmingen, San Diego, CA) for 1 h on ice in the dark and counterstained with the nuclear stain, DAPI (Blue). The cells were washed with FACS Buffer, centrifuged to a slide, examined and photographed by immunofluorescence microscopy as previously described (Ekong et al., 2009).

### Short interfering RNA (siRNA)

The expression of TLR4, MyD88, NF-κB, and Caspase-1 were blocked by transfection with siRNA (Thermo Fisher Scientific, Dharmacon Products) using the manufacturer's protocol. DCs were transiently transfected with Lipofectamine RNAiMAX Reagent-Invitrogen (Catalog number: 13778075) (Thermo Fisher Scientific, Waltham, MA) and siRNA targeting TLR4, MyD88, NF-κB, or Caspase-1 for 48 h. In brief, for RNAiMAX-RNAi duplex complex formation, 50 pmol of siRNA or ON-TARGET plus Non-targeting siRNA #1 (S-siRNA) (Dharmacon) controls were added to 500 μl of Opti-MEM I Reduced Serum Medium (Thermo Fisher Scientific, Waltham, MA) in each well of a six-well plate (Thermo Fisher Scientific)., Next, RNAiMAX (7.5 μl) was incubated with the diluted RNAi molecules in each well at room temperature. After 20 min, the DCs (5 × 10^5^) were then plated in 2.5 ml of complete RPMI medium (without antibiotics) per well. All transfections were carried out in triplicate. Forty-eight hours after addition of the siRNA or S-siRNA, DCs were treated with Pmp18.1 in RPMI-1640 medium containing 10% FBS and incubated for 48 h at 37°C/CO_2_. Protein extracts from each treated group was subjected to SDS-PAGE and Western immunoblotting analysis and proteins were detected using rabbit monoclonal antibodies (mAb) to Toll-like Receptor 4 (D8L5W, #14358), MyD88 (D80F5, #4283) and NF-κB p65 (D14E12, #8242), and Caspase-1 antibody (#2225) obtained from Cell Signaling Technology (Danvers, MA). Culture supernatants from siRNA treated and untreated DCs were analyzed for the amount of secreted IL-1β by the Bio-Plex cytokine ELISA assay according to the manufacturer's instructions. The mean (±*SD*) of triplicate cultures were calculated and expressed in pg/ml as means and standard deviations. The experiment was repeated twice.

### Statistical analysis

The GraphPad Prism package (GraphPad Software, Inc. La Jolla, CA, USA) on a MAC computer was used for statistical analyses. The Student's *t*-test was used to determine differences between two groups and differences between more than two groups were evaluated by one-way ANOVA. Differences were considered to be statistically significant when *P* values were < 0.05 or < 0.01.

## Results

### Purification of recombinant Pmp18.1

**Figure 1:**
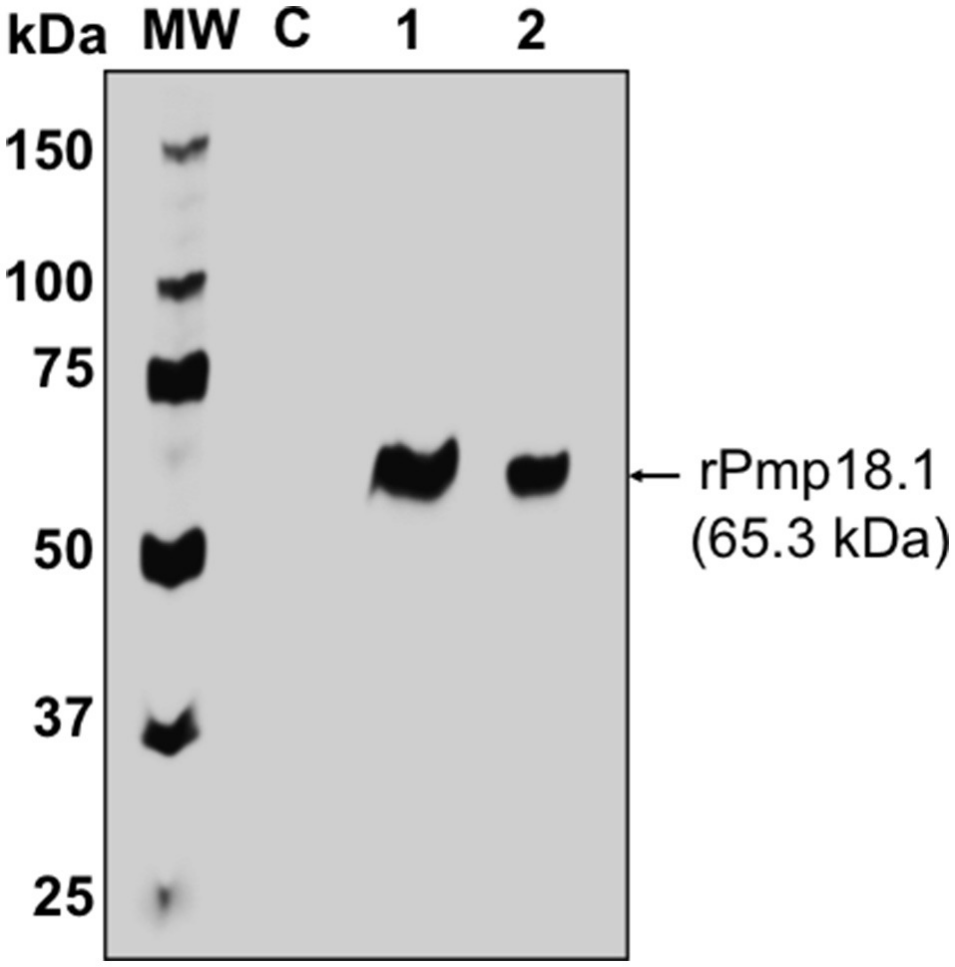
Detection of recombinant Pmp18.1 by Western immunoblotting analysis. The gene fragment *Pmp18.1* (encoding residues 122–560 of mature Pmp18D) was cloned in-frame and downstream of the *TrxA* gene fragment (encoding the thioredoxin tag sequence) of plasmid pET-32a(+), and the recombinant gene was expressed under the control of an IPTG-inducible T7 promoter. The rPmp18.1 was purified as a His-tag fusion protein using the Ni-NTA Purification System as described in the materials and methods. Samples were reduced and boiled prior to loading and electrophoresis on a 15% SDS-PAGE gel and the Pmp18.1 protein was detected by immunoblotting analysis using rabbit polyclonal antibody to Pmp18D. Lane C, *E. coli* BL21 (DE3) containing the plasmid vector, pET-32a; lane 1, rPmp18.1 (200 ng); lane 2, rPmp18.1 (100 ng). MW, molecular weight standards, and numbers to the left are approximate molecular masses, in kilodaltons (kDa).

The Ni-NTA Purification System (Invitrogen, Carlsbad, CA) was used for the purification of recombinant Pmp18.1. Endotoxin level was determined to be < 0.05 EU/mg protein. Protein concentration was estimated using the Pierce™ BCA Protein Assay Kit (Thermo, Illinois), adjusted to 500 μg/ml and stored at −80°C. The purified recombinant Pmp18.1 was detected by SDS-PAGE and immunoblotting analysis using purified rabbit anti-Pmp18D polyclonal antibody. The antibody reacted specifically with the purified N-terminal Pmp18D fragment (Pmp18.1) as indicated by a specific band at an approximate molecular mass of 65 kDa (Figure 1).

### Recombinant Pmp18.1 induces BMDC surface activation marker expression

**Figure 2:**
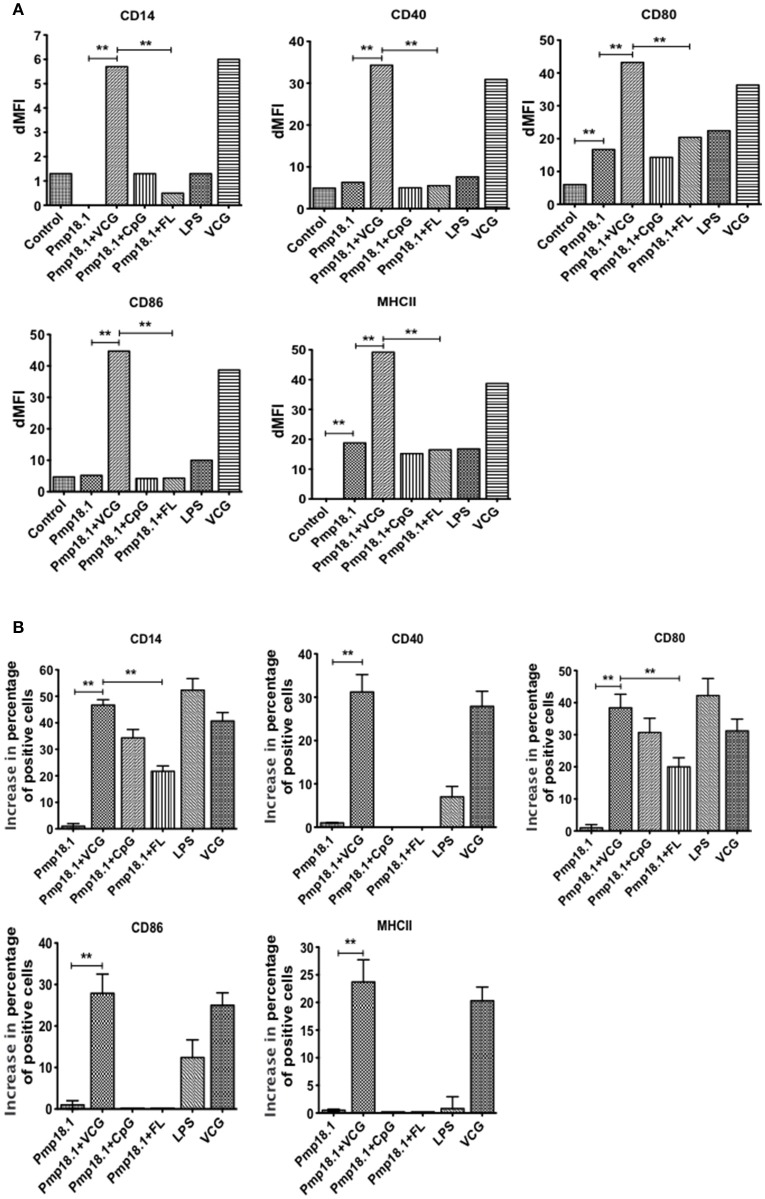
BMDC surface activation marker expression using FACS analysis. Immature BMDCs were obtained from femurs and tibias of C57BL/6J mice. BMDCs were isolated from mice by established procedures as described previously (Inaba et al., 2001). After 7 days of culture, cells were harvested, counted, and seeded again at 1 × 10^6^ cells per milliliter. Harvested cells were pulsed for 24 h with Pmp18.1 in the presence or absence of VCG, CpG, or FL adjuvants, or LPS (positive control) or medium (negative control) stained with conjugated monoclonal antibodies against CD14, CD40, CD80, CD86, 1Ab, or isotype-matched controls, and quantified in triplicate by flow cytometry. Gating was on CD11c+ DCs and the cells were identified based on light-scatter properties of DCs and CD11c expression (not shown). The data shows **(A)** a comparison of the differences between the mean fluorescence intensity (MFI) of stained samples and that of unstained samples (dMFIs) and **(B)** the percentage increase in the number of CD11c+ cells positive for the indicated markers. Data are from one representative experiment of three independent assays performed in duplicates with similar results. Statistical analyses were performed with GraphPad Prism. The statistical significance difference between two groups was evaluated by Student's *t*-test and between more than two groups by the one-way analysis of variance (ANOVA). Differences were considered to be statistically significant at ^**^p < 0.01.

We investigated the ability of Pmp18.1 to induce the maturation of BMDCs using LPS as a positive control. The data in Supplementary Figure 1 shows the mean fluorescence intensity (MFI) of staining of cell surface molecules. Figure 2A the dMFIs (the difference between the MFI of stained samples and the MFI of unstained samples) of surface molecules and (Figure 2B) the increase in the percentage of CD11c+ cells positive for the indicated markers over that induced by Pmp18.1. The results showed that except for CD80 and MHC II, DCs expressed levels of costimulatory molecules that were not significantly higher (*p* > 0.05) than those of unstimulated cells when stimulated with Pmp18.1 or LPS (positive control). Treatment of DCs with Pmp18.1 in combination with VCG or VCG alone significantly (*p* < 0.05) increased the surface expression of all the cellular markers over that of Pmp18.1 alone. On the other hand, combination of Pmp18.1 with CpG or FL did not significantly increase the expression of cellular markers compared to Pmp18.1 alone (Figure 2A). Furthermore, treatment of DCs with Pmp18.1 in combination with VCG or VCG alone showed a significant increase in the percentages of CD11c+ cells positive for the indicated markers (*p* < 0.05) compared to treatment with Pmp18.1 alone (Figure 2B). These results demonstrate that Pmp18.1 induces maturation of DCs *in vitro* and suggest its potential ability to induce an immune response when administered in an appropriate vaccine formulation.

### *C. abortus* Pmp18.1 stimulates the upregulated expression of TLRs *in Vitro*

**Figure 3:**
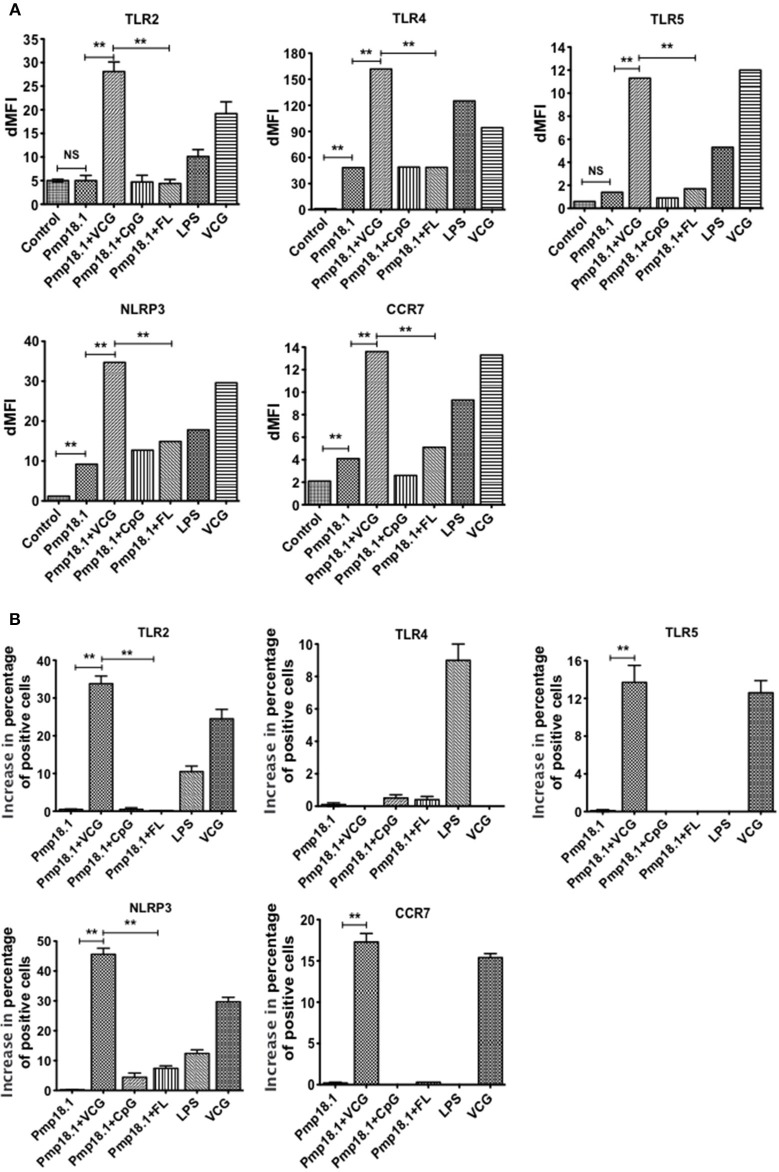
Activation of TLR and NLRP3 engagement by BMDCs stimulated with Pmp18.1. Harvested cells were pulsed for 24 h with Pmp18.1 in the presence or absence of VCG, CpG, or FL adjuvants, or LPS (positive control) or medium (negative control) stained with conjugated monoclonal antibodies against TLRs 2, 4, 5, CCR7, and NLRP3 inflammasome or isotype-matched controls, and quantified in triplicate by flow cytometry. Gating was on CD11c+ DCs and the cells were identified based on light-scatter properties of DCs and CD11c expression (not shown). The data shows **(A)** the dMFIs (the difference between the MFI of stained samples and the MFI of unstained samples) and **(B)** the percentage increase in the number of CD11c+ cells positive for the indicated receptors over that induced by Pmp18.1 alone. Data are from one representative experiment of three independent assays performed in duplicates with similar results. Statistical analyses were performed using the Student's *t*-test for differences between two groups or one-way ANOVA for differences between more than two groups. Statistically significant differences between treatment groups was evaluated at (^**^p < 0.01). NS indicates not significant.

We evaluated the ability of DCs pulsed with *C. abortus* Pmp18.1 with and without VCG, CpG, or FL to express TLRs *in vitro*. The data in Supplementary Figure 2 shows the MFI of staining of TLRs and NLRP3. Figure 3 shows the dMFIs (the difference between the MFI of stained samples and the MFI of unstained samples) of TLRs and (Figure 3B) the increase in the percentage of CD11c+ cells positive for the indicated TLRs over that induced by Pmp18.1. The data shows that DCs expressed significantly higher (*p* < 0.05) levels of TLR4, NLRP3, and CCR7 when stimulated with Pmp18.1 alone or in combination with either CpG or FL compared with unstimulated cells (Figure 3B). Interestingly, stimulation of DCs with Pmp18.1 in combination with VCG or treatment with VCG alone resulted in the upregulated expression of TLR2, TLR4, TLR5, NLRP3, and CCR7. Aside from TLR4, the DC expression of these receptors was significantly higher (*p* < 0.05) when stimulated with Pmp18.1 in combination with VCG than when combined with CpG or FL (Figure 3B). As expected, stimulation with LPS significantly (*p* < 0.05) increased the DC expression of TLR4. Treatment of DCs with Pmp18.1 in combination with VCG but not CpG or FL showed a significant increase in the percentages of cells positive for all the indicated receptors (*p* < 0.05) over that induced by Pmp18.1 alone (Figure 3C).

### Recombinant Pmp18.1 induces the DC secretion of proinflammatory cytokines

**Figure 4:**
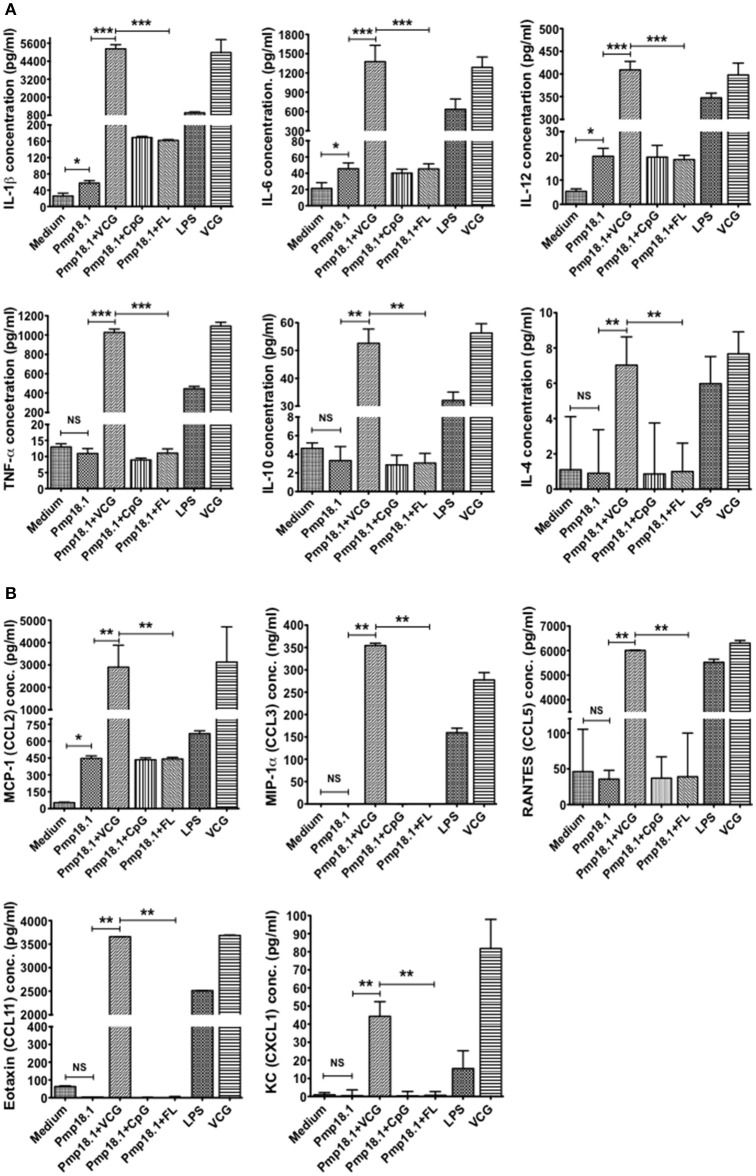
Activation of cytokine production by Pmp18.1-stimulated BMDCs. DCs were pulsed *ex vivo* with Pmp18.1 in the presence or absence of VCG, CpG or FL, or LPS (positive control) or medium (negative control) for 24 h. Culture supernatants were collected and assayed for Th1/Th2 cytokines and chemokines by cytokine ELISA using the Bio-Plex cytokine assay kit in combination with the Bio-Plex Manager software. The concentration of the cytokines **(A)** or chemokines **(B)** in each sample was obtained by extrapolation from a standard calibration curve generated simultaneously. Data are shown as the mean values (± *SD*.) for triplicate cultures for each experiment. The results are from two independent experiments. Statistical analyses were performed using the Student's *t*-test for differences between two groups or one-way analysis of variance ANOVA for differences between more than two treatment groups. Differences were considered to be statistically significant at ^*^p < 0.05 or ^**^p < 0.01 or ^***^p < 0.001. NS indicates not significant.

The ability of *C. abortus* Pmp18.1 with and without VCG, CpG, or FL to induce the production of proinflammatory cytokines in 48-h DC culture supernatants was also evaluated. Pmp18.1 stimulated the DC secretion of significantly higher (*p* < 0.05) levels of IL-1β, IL-6, and IL-12p70 and MCP-1 (CCL-2) cytokines compared with unstimulated cells (Figures 4A,B). When combined with VCG, Pmp18.1 also induced the secretion of significantly higher (*p* < 0.01) levels of TNF-α and IL-10 as well as the chemokines, MIP-1a (CCL3), RANTES (CCL5), Eotaxin (CCL1), and KC (CXCL1) compared to those pulsed with Pmp18.1 plus CpG or FL (Figures 4A,B). Also, culture with LPS induced the DC secretion of significantly higher (*p* < 0.05) levels of cytokines and chemokines compared to controls (Figure 4). These results demonstrate the potency of VCG in augmenting the Pmp18.1-induced DC TLR expression and cytokine secretion.

### VCG enhanced the rPmp18.1-induced proliferation of naïve CD4+ T cells

**Figure 5:**
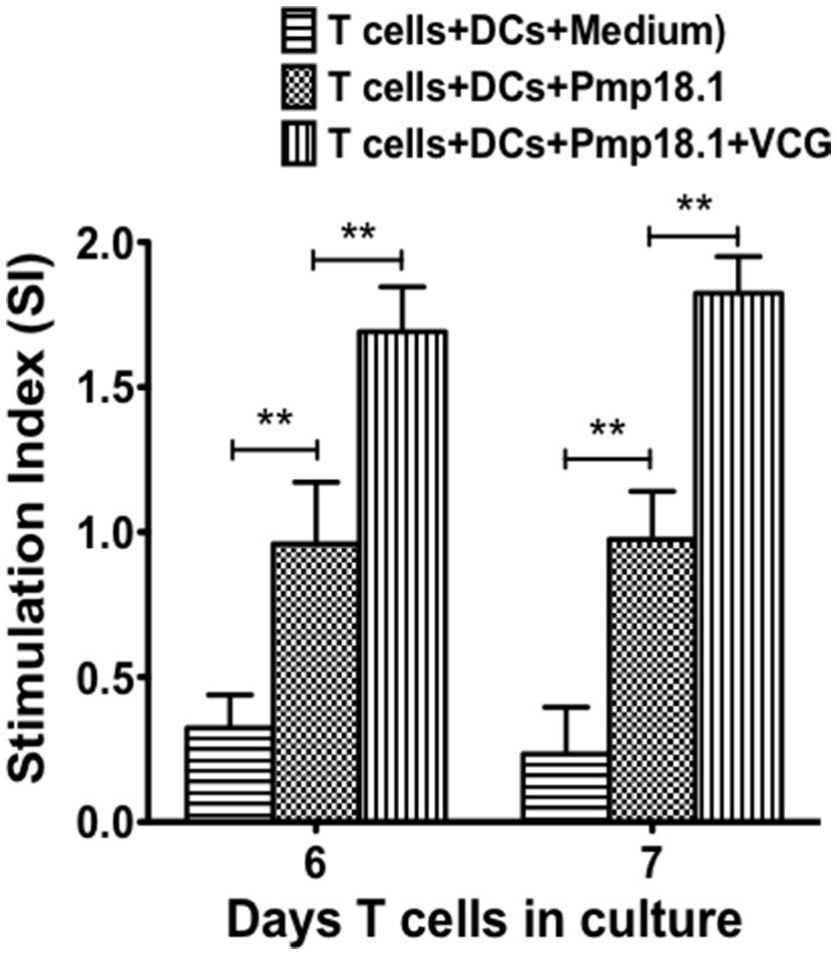
Pmp18.1-induced proliferation of naïve CD4+ T cells. Splenic naïve CD4^+^ T cells were cultured with BMDCs (2 × 10^5^ cells/well) or BMDCs previously pulsed for 24 h with VCG (100 μg) in the presence or absence of Pmp18.1 (10 μg/ml) for 7 days. The antigen-specific proliferative response was determined using the BrdU incorporation assay; incorporation was detected by addition of ABTS substrate and the optical density was read at 405 nm using a scanning multi-well spectrophotometer. Results are expressed as the stimulation index (SI), the ratio between absorbance values of stimulated and non-stimulated cells and the bars represent the mean and *SD*. of three independent experiments. Statistically significant differences between DCs alone and treatment groups and between the treatment groups was evaluated at (^**^p < 0.01) using the Student's *t*-test.

We investigated if rPmp18.1 could mediate the *in vitro* proliferation of naïve CD4+ T cells in the presence or absence of VCG. Thus, BMDC were cultured with naïve CD4+ T cells for 7 days in the presence of Pmp18.1 alone or Pmp18.1 plus VCG and T cell proliferation was assessed by the 5-Bromo-2′-deoxy-uridine cell proliferation assay. DCs cultured with T cells in the absence of Pmp18.1 served as control. The effect of VCG on antigen-specific T cell proliferation was determined by analyzing the ratio between absorbance values of stimulated and non-stimulated CD4+ T cells [stimulation index (SI) values] following stimulation with Pmp18.1 in the presence or absence of VCG. The data shows the derived SI values (Figure 5). The results showed naïve CD4 T cells incubated with Pmp18.1 proliferated significantly higher (*p* < 0.01) compared to unstimulated cells on days 6 and 7 following stimulation. Moreover, VCG enhanced the Pmp18.1-mediated proliferation of the naïve CD4 T cells (Figure 5). The results indicate that VCG could enhance the ability of BMDC to present Pmp18.1 antigen to naïve CD4+ T cells *in vitro*.

### Pmp18.1 activates the expression of MyD88, NF-κB, and caspase-1 inflammasome in DCs

**Figure 6:**
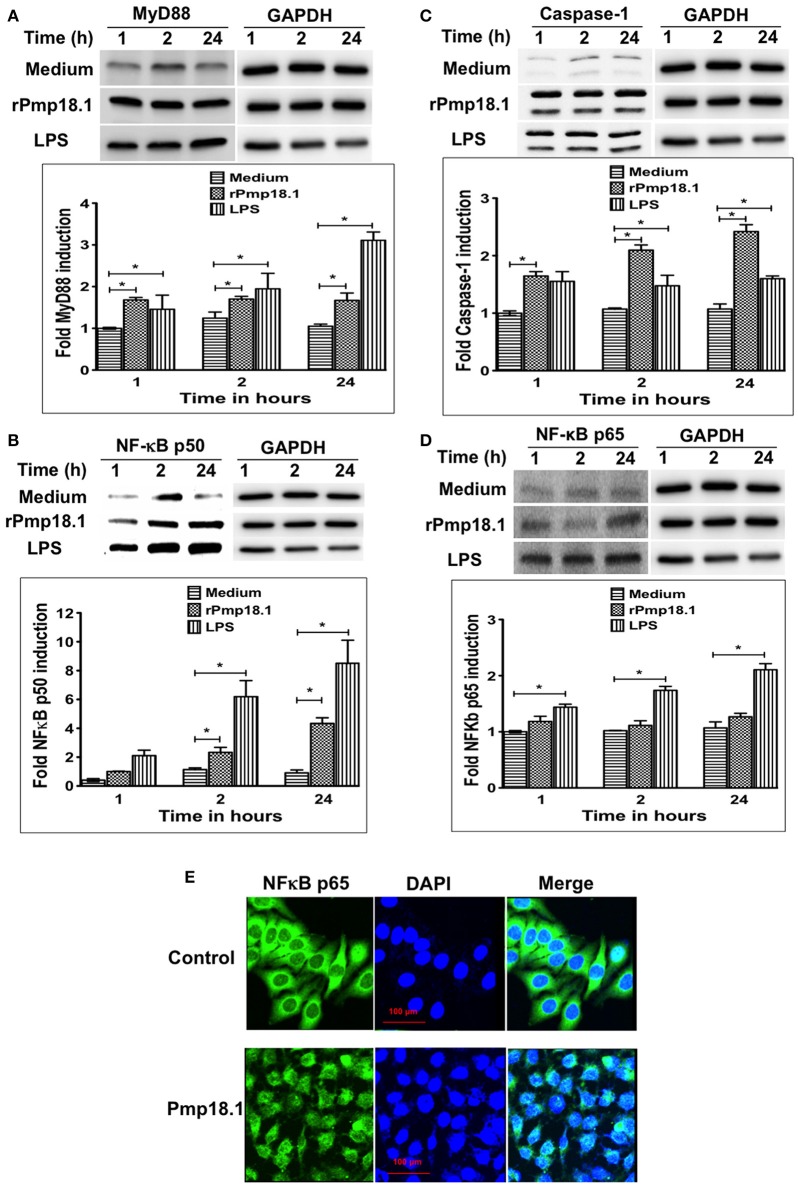
Pmp18.1 activates MyD88, NFκB and Caspase-1 expression in BMDCs. Cell lysates were prepared from BMDCs incubated with Pmp18.1 (10 μg/ml) for 1, 2, or 24 h. Protein extracts (40 μg/well) were separated by SDS-PAGE and detected by immunoblotting analysis incorporating the Pierce™ ECL Western Blotting Substrate using antibodies to the corresponding proteins. The proteins bands were visualized using the ImageQuant LAS-4000 imaging system. Protein levels, normalized to GAPDH, were then quantified using Image J software. The data shows the expression levels and fold induction of TLR4 **(A)**, MyD88 **(B)**, NF-κB p50 **(C)**, and Caspase-1 **(D)**. Each experiment was repeated at least two times with similar findings. For NF-κB p65 nuclear migration, BMDCs were incubated for 24 h with rPmp18.1 and nuclear NF-κB p65 was detected by confocal microscopy using a FITC conjugated monoclonal antibody to NF-κB p65 (clone D14E12, Cell Signaling Technology). **(E)** NF-κB p65 unit was stained in green, and nuclei were visualized by DAPI counterstain (blue). Note the diffuse distribution of NF-κB p65 (green) in the nucleus. The statistical significance difference between two groups was evaluated by Student's *t*-test and between more than two groups by the one-way analysis of variance (ANOVA). Differences were considered to be statistically significant at ^*^p < 0.05.

Western blot analysis of lysates from DCs stimulated with rPmp18.1 showed increasing expression of MyD88 (Figure 6A) and NF-κB p50 (Figure 6B) at 1, 2, or 24 h post-stimulation. Expression levels of MyD88 and NF-κB in Pmp18.1-treated DCs were significantly higher (*p* < 0.05) than those of the non-treated group (medium control) at all time points post-stimulation. Also, LPS-stimulated DCs expressed high levels of the proteins at all time points. As DCs expressed high levels of NLRP3 inflammasome and secreted IL-1β following stimulation with Pmp18.1, we investigated if rPmp18.1 could also activate the expression of Caspase-1 in treated DCs. The result showed high expression levels of Caspase-1 comparable to that induced by LPS at all time points post-stimulation (Figure 6C). As expected, GAPDH (housekeeping protein) was detected in equal amounts in cell lysates of treated and untreated (culture control) DCs at all time points (Figures 6A–C). The results indicate that *C. abortus* Pmp18.1 activated the cytoplasmic expression of MyD88 and NF-κB in treated DCs.

### Pmp18.1 activates the nuclear translocation of NF-κB p65 in treated DCs

In addition to the cytoplasmic expression of NF-κB, Western blot analysis also showed the migration of NF-κB p65 protein subunit from the cytoplasm into the nucleus of Pmp18.1 treated DCs as indicated by the detection of NF-κB p65 with increasing intensities in nuclear extracts at 1, 2, or 24 h post-stimulation (Figure 6D). NF-κB p65 and GAPDH were detected in nuclear extracts of DCs treated with LPS at all the time points evaluated. In addition, GAPDH (housekeeping protein) was detected in equal amounts in cell lysates of treated and untreated (culture control) DCs at all time points (Figure 6D). Confocal microscopy analysis confirmed the nuclear translocation of NF-κB p65 as indicated by the high intensity of p65 staining (green) in the nucleus of DCs treated with Pmp18.1 compared to untreated cells (Figure 6E). The DAPI stained nuclei of both treated and untreated cells appeared blue. These results confirm the ability of Pmp18.1 to activate the translocation of NF-κB p65 from the cytoplasm to the nucleus of treated DCs.

### Short interfering RNA (siRNA) targeting inhibits IL-1β secretion by Pmp18.1-stimulated BMDCs

**Figure 7:**
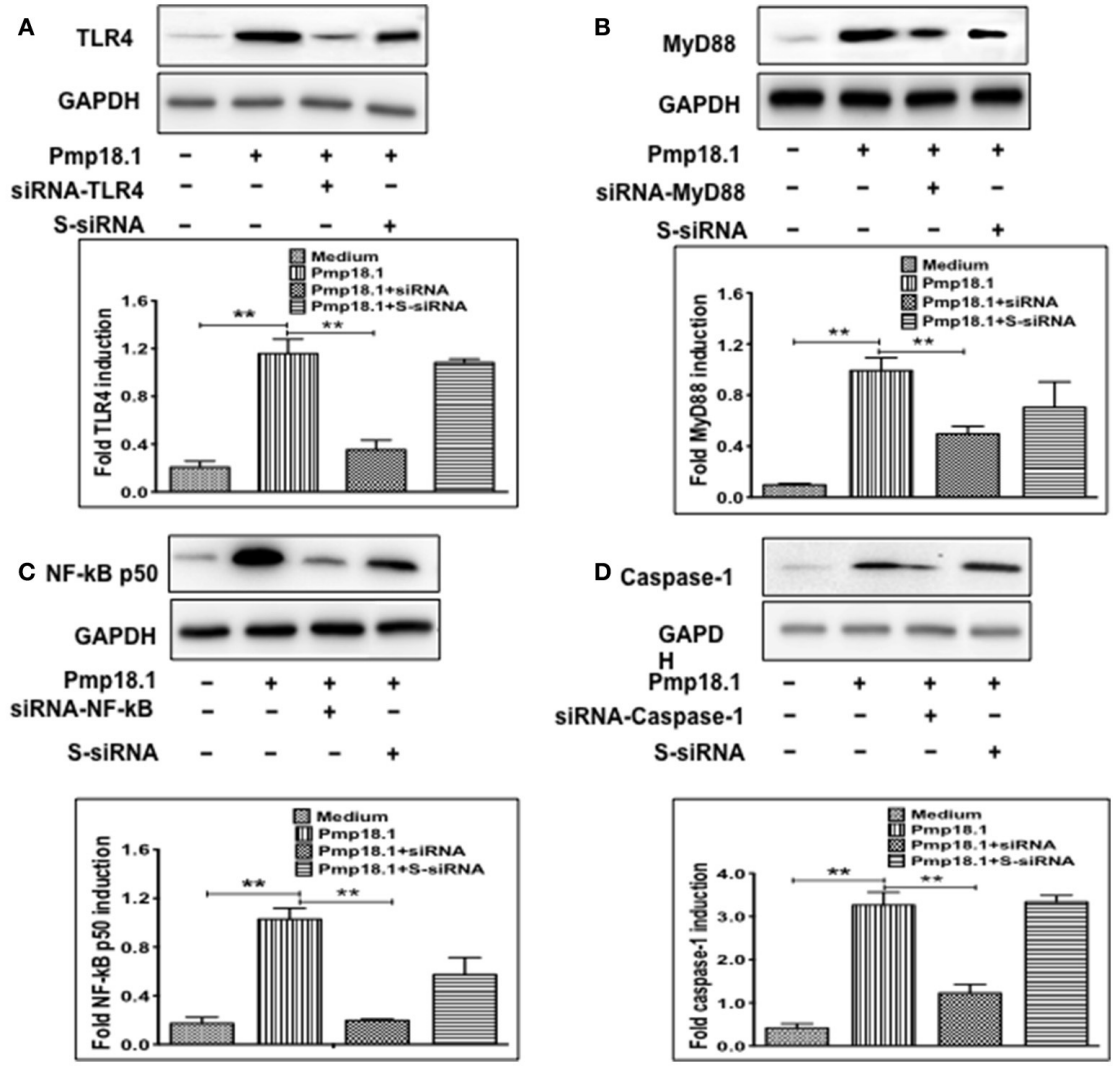
siRNA targeting reduces TLR4, MyD88, NFκB, and Caspase-1 expression in DCs. BMDCs were separately transfected with siRNA targeting TLR4, MyD88, NF-κB, or Caspase-1 for 48 h before stimulation with Pmp18.1 for 24 h. Protein extracts were subjected to SDS-PAGE and proteins were detected by Western immunoblotting analysis using rabbit monoclonal antibodies (mAb) to TLR 4 (D8L5W), MyD88 (D80F5), and NF-κB p65 (D14E12), and mouse mAb to Caspase-1 (14F468). The proteins bands were visualized using the ImageQuant LAS-4000 imaging system. Protein levels, normalized to GAPDH, were then quantified using Image J software. Each experiment was repeated at least twice. The results are from two independent experiments. Statistical analyses were performed using the Student's *t*-test. Statistically significant differences between rPmp18.1 stimulated and unstimulated DCs (Medium) and between siRNA-treated and scrambled siRNA-treated (S-siRNA) cells were evaluated using the Student's *t*-test and were considered to be statistically significant at (^**^p < 0.01).

**Figure 8:**
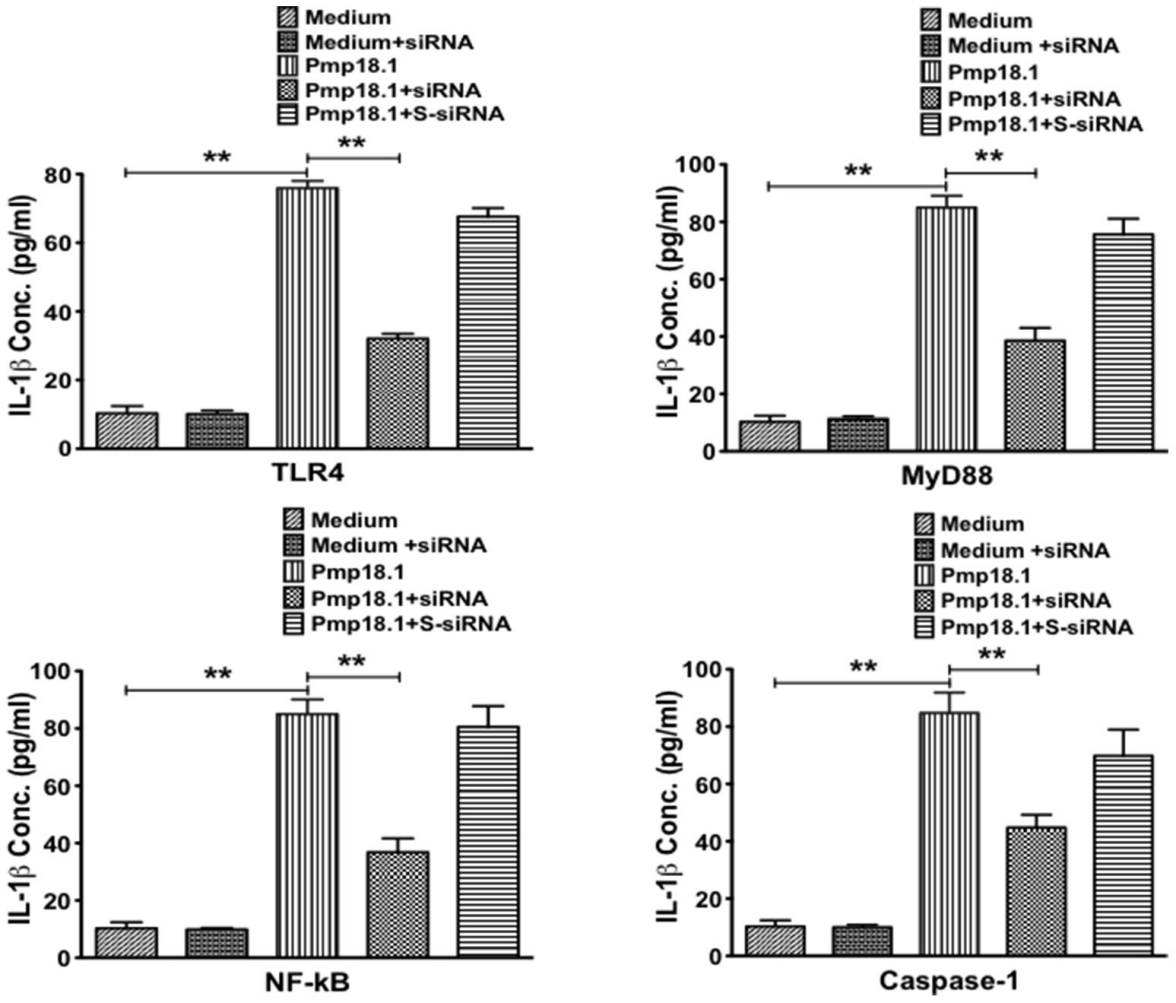
siRNA targeting of TLR4, MyD88, NFκB, and Caspase-1 inhibits DC secretion of IL-1β. BMDCs were separately transfected with siRNA targeting TLR4, MyD88, NF-κB, or Caspase-1 for 48 h before stimulation with Pmp18.1 for 24 h. Culture supernatants were collected and assayed for magnitude of IL-1β by cytokine ELISA using the Bio-Plex cytokine assay kit in combination with the Bio-Plex Manager software. The concentration of IL-1β in each sample was obtained by extrapolation from a standard calibration curve generated simultaneously. Data are shown as the mean values (± *SD*.) for triplicate cultures for each experiment. The statistical significance difference between two treatment groups was evaluated by Student's *t*-test and between more than two treatment groups by the one-way analysis of variance (ANOVA). Differences were considered to be statistically significant at ^**^p < 0.01.

We further investigated if inhibiting Pmp18.1-mediated activation of TLR4, MyD88, NF-κB p50, and Caspase-1 proteins using siRNA would influence the DC production of IL-1β. Results from the Western immunoblotting analysis showed that targeting TLR4, MyD88, NF-κB p50, and Caspase-1 mRNA with siRNA significantly reduced their expression levels compared to levels in DCs treated with Pmp18.1 in the absence of siRNA (Figure 7). The reduced protein levels in DCs previously treated with siRNA resulted in a significant decrease in the magnitude of IL-1β cytokine secretion associated with Pmp18.1 treatment (Figure 8). These results suggest the involvement of TLR4, MyD88, NF-κB p50, and Caspase-1 signaling in the Pmp18.1-induced IL-1β production.

## Discussion

The immune response stimulated by a vaccine is largely dependent on the choice of the immunogen. Pmp18D is an immunogenic polymorphic outer membrane protein that plays an important role in *C. abortus* pathogenesis. We recently identified an N-terminal fragment of the *C. abortus* Pmp18D protein (designated Pmp18.1) as a possible vaccine antigen. Previous studies demonstrated that the immunogenicity of an antigen could be predicted on the basis of its capacity to induce immune responses *in vitro*, using normal peripheral blood mononuclear cells (PBMCs) and cytokine-induced DCs (Keogh et al., 2001). DCs are a group of professional APCs, which initiate and control antigen-specific immune responses and have been used for the screening and selection of vaccine antigens (Karunakaran et al., 2015). Effective induction of a Th1-specific immune response is contingent on the capacity of DCs to present antigen in the context of major histocompatibility complex molecules to T cells, provide costimulation via CD80 (B7.1) or CD86 (B7.2) molecules, and secrete IL-12 that polarizes the responding T cells to a Th1 phenotype (Banchereau and Steinman, 1998; Banchereau et al., 2000). Thus, to evaluate the vaccine potential of the newly identified *C. abortus* Pmp18.1, we first investigated its ability to induce innate immune responses in dendritic cells *in vitro*. Culture of rPmp18.1 with BMDC resulted in the upregulated DC expression of MHC II and co-stimulatory molecules as well as TLR4 and intracellular NLRP3. These findings confirm the results of our previous studies reporting that inactivated chlamydial whole cell and subunit antigens stimulate the *in vitro* maturation of DCs (Eko et al., 2014; Pan et al., 2015). Engagement of CD40 on APCs has been demonstrated to lead to the up-regulated expression of MHC-II and CD80 and CD86n and production of inflammatory cytokines, such as TNF-α and IL-12 (Diehl et al., 2000). A previous study reported that NLRs, which are a subset of cytosolic PRRs, are essential for detecting PAMPs and induction of innate immune responses (Tang et al., 2012). The binding of TLRs and NLRs to microbial PAMPs results in activation of DCs and subsequent secretion of proinflammatory cytokines (O'donnell et al., 2014). Furthermore, activation of the NLR inflammasome causes the stimulation of the caspase-1 pathway, which influences the induction of innate and acquired immunity (O'donnell et al., 2014). The NLRP3 inflammasome and Caspase-1 have previously been reported to contribute to IL-1β processing and secretion by neutrophils (Winkler et al., 2017) (Karmakar et al., 2015). The upregulated expression of NLRP3 by rPmp18.1-treated DCs suggests the involvement of the inflammasome in IL-1β processing and secretion by DCs.

Since proinflammatory cytokines, such as IL-1β are involved in shaping the inflammatory response against pathogens, which is essential for effective adaptive immune induction and clearance of infection (Abdul-Sater et al., 2010), we examined DC cultures stimulated with rPmp18.1 for the amount and profile of proinflammatory cytokines secreted. Stimulation of BMDCs with rPmp18.1 resulted in secretion of IL-1β, IL-12, IL-6, and MCP-1 (CCL2). IL-12 is responsible for T cell proliferation and Th1 immune priming (Macatonia et al., 1995) and IL-6 is essential for B cell activation and antibody production (Macatonia et al., 1989; Mayer et al., 2014). CCL2 has been shown to promote DC migration to lymph nodes and subsequent maturation (Ouwehand et al., 2010). In a previous study, we showed that UV-inactivated chlamydial elementary bodies activated the secretion of IL-12 and TNF-α following co-culture with BMDCs (Eko et al., 2014). Also, a VCG-delivered MOMP vaccine activated DCs to secrete IL-12, TNF-α, and GM-CSF (Ekong et al., 2009). It has been documented that following antigen uptake, activated DCs stimulate the proliferation and differentiation of T cells into functional helper and effector cells (Ueno et al., 2011). We used the DC-T cell assay, which is a useful and sensitive assay for assessing T cell proliferation, to evaluate the ability of rPmp18.1 to stimulate T cell proliferative responses that could subsequently lead to induction of protective immunity. Previously, we showed that UV-inactivated chlamydial EBs and a purified *C. abortus* antigen induced the proliferation of naïve and immune T cells (Eko et al., 2014; Pan et al., 2015). CD4+ T cells isolated from naïve mice proliferated significantly in response to stimulation with rPmp18.1, indicating the ability of BMDC to efficiently present rPmp18.1 antigen to naïve CD4+ T cells *in vitro* that is likely to lead to induction of protective immunity.

One of the characteristics of a vaccine adjuvant is the ability to enhance protective immunity when co-delivered with antigens either alone or in combination with other adjuvants. In this regard, a combination of CpG and the B subunit of cholera toxin (CTB) was found to augment protective immunity of purified chlamydial MOMP (Cheng et al., 2009). In another study, CpG was reported to be the most effective adjuvant in enhancing the protective immunity of chlamydial MOMP when compared to several TLR ligands (Cheng et al., 2011). Recently, a combination of CpG and Pam2CSK4 was shown to elicit a Th2 immune response while the combination of CpG and Montanide ISA 720 elicited a Th1 response (Cheng et al., 2013). FL, which has been reported to induce the proliferation of dendritic cells, was demonstrated to enhance the immune responses of a *Streptococcus pneumoniae* antigen when used in combination with CpG (Fukuyama et al., 2011; Kataoka et al., 2011). Moreover, this combination also induced the proliferation of DCs and influenza-specific protective immunity to influenza virus hemagglutinin in aged mice (Asanuma et al., 2012). In this study, we investigated the immunomodulatory impact of VCG, in comparison with the more established Th1-promoting adjuvants CpG and FL, on the rPmp18.1-mediated innate immune activation. Remarkably, VCG, but not CpG or FL enhanced the rPmp18.1-stimulated expression of DC maturation markers and CCR7. CCR7 (CD197), which is expressed by mature DCs, is critical in the interaction of mature antigen presenting DCs with naïve T-cells and is a prerequisite for the induction of antigen-specific T-cell immunity (Jørgensen et al., 2017). Also, VCG, but not CpG or FL increased the repertoire of TLRs expressed as well as enhanced the magnitude and number of proinflammatory cytokines secreted and proliferation of naïve T cells. We previously showed that VCG could enhance the ability of inactivated chlamydial elementary bodies and a purified recombinant *C. abortus* outer membrane protein to stimulate the maturation of BMDCs leading to increased secretion of proinflammatory cytokines and T cell proliferation (Eko et al., 2014; Pan et al., 2015). VCG was found to be superior to the combination of CpG and FL in enhancing DC maturation and secretion of proinflammatory cytokines (Pan et al., 2015). VCG has also been shown to be an effective delivery vehicle and immunomodulator, augmenting the immune responses and protection of cloned chlamydial antigens (Eko et al., 2004, 2011a,b; Pais et al., 2017). In addition to increasing the magnitude and profile of proinflammatory cytokines, VCG also enhanced the ability of DCs to present rPmp18.1 antigen to naïve CD4+ T cells. This VCG-mediated immune enhancement is critical as innate immunity has been demonstrated to play a vital role in primary *C. abortus* infection and induction of specific immunity through T-cell recruitment and cytokine secretion (Buendia et al., 2004).

Previous studies have demonstrated that specific PRRs, such as TLRs, are stimulated upon recognition of various bacterial PAMPs leading to induction of immune responses mainly triggered via activation of the transcription factor, NF-κB (Joyee and Yang, 2008; Hodgson and Wan, 2016). Since DCs expressed high levels of TLR4 when stimulated with Pmp18.1 and given that MyD88 and NF-κB are the classical downstream signaling pathways of TLRs, we investigated if rPmp18.1 could activate the expression of MyD88 and NF-κB in treated DCs. Indeed, treatment of DCs with Pmp18.1 activated the protein expression of MyD88, NF-κB p50, and Caspase-1 and the nuclear translocation of NF-κB p65. Furthermore, as treatment of DCs with Pmp18.1 induced the secretion of high levels of the proinflammatory cytokines IL-1β, IL-12, and IL-6 and activated the expression of TLR4, MyD88, NF-κB p50, and Caspase-1, we investigated whether inhibiting Pmp18.1-mediated activation of these proteins using siRNA would influence the DC production of IL-1β. IL-1β was chosen as a representative of the proinflammatory cytokines elevated following treatment of DCs with Pmp18.1 to evaluate the effect of treatment with siRNA. We demonstrated that siRNA targeting TLR4, MyD88, NF-κB p50, and Caspase-1 mRNA in DCs significantly reduced their expression levels resulting in decreased IL-1β cytokine secretion strongly suggesting the involvement of TLR4, MyD88, NF-κB p50, and Caspase-1 signaling in the Pmp18.1-induced IL-1β secretion. These results confirm our previous prediction that DC proinflammatory cytokine production may involve the TLR and MyD88 as well as the inflammasome and caspase pathways (Pan et al., 2015). Activation of NF-κB is initiated by the binding of NF-κB ligand to a PRR and signal transduction by MyD88, which leads to the IκB kinase (IKK) complex activation (Hayden and Ghosh, 2012). Phosphorylation of the IKK beta subunit (IKKβ) results in proteasomal degradation of IκB, which leads to the liberation and translocation of the activated NF-κB complex into the nucleus. Following nuclear translocation, the NF-κB complex binds to an array of κB sites in the genome resulting in the transcription of target genes, such as those regulating immune responses (Lenardo et al., 1989; Hayden and Ghosh, 2012). Regulation of the expression of pro-IL-1β and inflammasome-related protein genes results in the intracellular NLRP3 and apoptosis-associated speck-like protein containing CARD (ASC) expression (Omosun et al., 2015). Activation of Caspase-1 and subsequent secretion of mature IL-1β is the consequence of ASC/Caspase-1 complex formation with NLRP3 inflammasome.

In conclusion, we have demonstrated that *C. abortus* Pmp18.1 promoted the upregulated expression of molecules associated with DC maturation, T cell activation and differentiation. We also demonstrated that VCG significantly enhanced the innate immune responses elicited by rPmp18.1. Furthermore, TLR4 activation in rPmp18.1-pulsed DCs stimulated the MyD88/NF-κB/Caspase-1 and IL-1β pathways as confirmed by decreased IL-1β production in siRNA-mediated TLR4/MyD88/NF-κB/Caspase-1 knockdown DCs. Taken together, these data indicate that *C. abortus* Pmp18.1 induces IL-1β secretion by TLR4 activation through the MyD88, NF-κB and Caspase-1 signaling pathways and may be a potential *C. abortus* vaccine candidate. The vaccine potential of Pmp18.1 will subsequently be evaluated in an appropriate animal model, using VCG as an immunomodulator, following immunization and challenge.

## Author contributions

FE, CH and QP conceived and designed the research. QP, QZ, JC, RP, and SL performed the experiments. FE, QP and QZ analyzed the data; and FE and QP wrote the paper. All authors have read and approved the manuscript.

### Conflict of interest statement

The authors declare that the research was conducted in the absence of any commercial or financial relationships that could be construed as a potential conflict of interest.
