# Supplementary figures and images for: Chlamydia abortus Pmp18.1 Induces IL-1β Secretion by TLR4 Activation through the MyD88, NF-κB, and Caspase-1 Signaling Pathways
Source: Front Cell Infect Microbiol. 2017 Dec 18;7:514. doi: 10.3389/fcimb.2017.00514 (PMC5741698; doi:10.3389/fcimb.2017.00514)

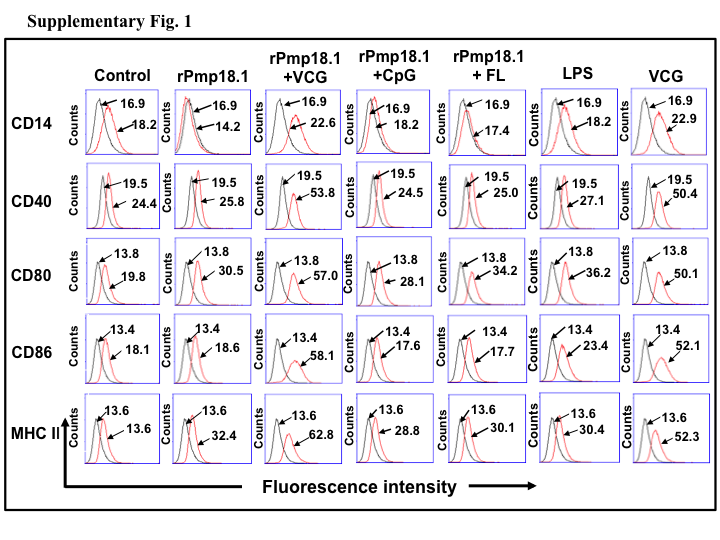

Supplement: Supplementary file 1 [file Image1.TIFF]

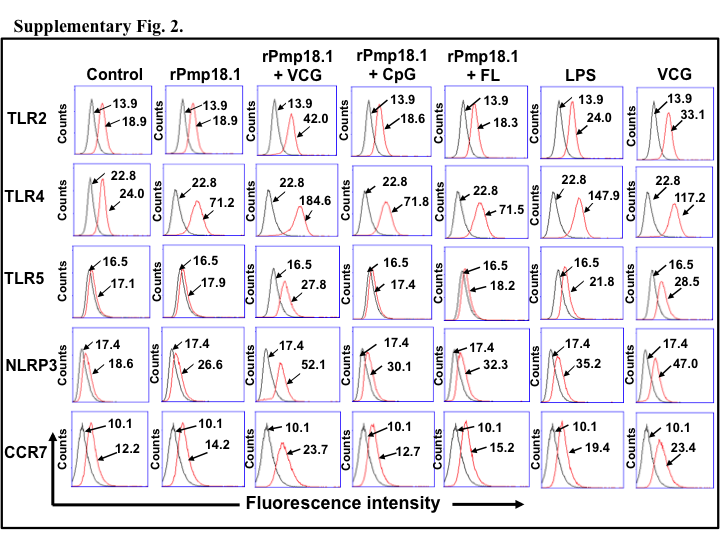

Supplement: Supplementary file 2 [file Image2.TIFF]
